# Antioxidant Status in Children with Neurogenic Bladder

**DOI:** 10.3390/children12060668

**Published:** 2025-05-23

**Authors:** Joanna Bagińska-Chyży, Agata Korzeniecka-Kozerska

**Affiliations:** Department of Pediatrics and Nephrology, Medical University of Białystok, 17 Waszyngton Str., 15-274 Białystok, Poland; agatakozerska@poczta.onet.pl

**Keywords:** neurogenic bladder, oxidative stress, total antioxidant status

## Abstract

Background: Pediatric neurogenic bladder (NB), often resulting from myelomeningocele, impairs bladder function due to disrupted neural control and is worsened by urinary retention, recurrent urinary tract infections, the absence of voluntary voiding, and additional sequelae of myelomeningocele, such as motor impairments, delayed colonic transit, and nutritional deficiencies. Oxidative stress arises from an imbalance between oxidant production and the body’s antioxidant defenses and is recognized as both a contributor to and a consequence of various pathological conditions. This study aims to assess the total antioxidant status (TAS) in NB patients, evaluate its impact on urinary antioxidants, and correlate the findings with the urodynamic parameters in NB patients compared to those in non-NB controls. Methods: This study included 29 patients with NB, who were compared with 57 non-NB individuals. The comparative analyses encompassed serum and urinary total antioxidant status normalized to creatinine (uTAS/creatinine) and renal function markers (creatinine, urea, uric acid, and the glomerular filtration rate [GFR]), as well as urodynamic findings. TAS was determined using the colorimetric ABTS method. Results: The patients with NB demonstrated a significantly lower serum TAS and elevated urinary TAS and uTAS/creatinine ratios in comparison to these values in the control group (*p* < 0.001). Furthermore, a positive correlation was observed between uTAS/creatinine and detrusor pressure at the maximum cystometric capacity, while a negative correlation was found between uTAS/creatinine and bladder wall compliance (r = 0.5, r = −0.68 respectively). Conclusions: The observed decrease in serum TAS and the increase in urinary TAS in NB may not only serve as evidence of an imbalance in antioxidant homeostasis but also suggest a potential contributory role to the deterioration of urodynamic function.

## 1. Introduction

Oxidative stress arises from an imbalance between oxidant production and the body’s antioxidant defenses and is widely acknowledged as both a primary etiological factor in and a secondary contributor to the progression of numerous pathological conditions. In certain diseases, such as radiation-induced toxicity and atherosclerosis, oxidative stress serves as a direct causative agent [1,2]. In contrast, in disorders including, e.g., cardiovascular, neurodegenerative diseases, type 2 diabetes mellitus, and osteoporosis, it plays a more secondary, yet still pivotal, role in exacerbating disease progression [3,4,5]. Particularly in urinary tract pathologies, oxidative stress is recognized as a central pathogenic mechanism, largely due to the bladder and kidneys’ heightened vulnerability to oxidative damage. It can modulate a range of intracellular signaling pathways and disrupt critical biological functions by inducing protein modifications, promoting inflammatory responses, and triggering apoptotic pathways [6,7,8]. Collectively, these alterations contribute to the acceleration of disease pathogenesis and the intensification of clinical manifestations.

Pediatric neurogenic bladder (NB) is a significant dysfunction in bladder control resulting from impaired neural innervation, most frequently associated with myelomeningocele (MMC). Oxidative stress has been implicated from the earliest stages of disease development. Given the multifactorial nature of the pathogenesis of neural tube defects, folic acid deficiency remains one of the most well-established contributing factors. During early embryonic development, the neural tube is especially vulnerable to oxidative damage because it is a rapidly growing and differentiating structure. Evidence suggests that impaired activity of key antioxidant enzymes—such as glutathione peroxidase, glutathione S-transferase, and catalase—may play a critical role in the etiology of MMC, particularly by exacerbating folate-related oxidative stress pathways [9,10].

Oxidative stress may also influence the postnatal clinical course of MMC by contributing to secondary complications such as chronic inflammation, neurodegeneration, poor wound healing, and renal dysfunction. Immediately following delivery, neonatal care focuses on preventing infection and protecting the exposed neural tissue, often necessitating prompt surgical closure of the spinal defect [11]. Postoperatively, individuals with MMC often experience a variety of chronic complications, the severity of which is largely determined by the anatomical level and extent of the spinal lesion. Hydrocephalus is common and often requires ventricular shunting. Neurological impairments may include lower limb motor and sensory deficits, orthopedic deformities, and varying degrees of bowel and bladder dysfunction, most notably NB. It is also a risk factor for recurrent urinary tract infections (UTIs), reflux nephropathy, urinary retention, and the absence of voluntary voiding. Throughout infancy and childhood, continuous monitoring of renal function, periodic urodynamic evaluations, and the implementation of clean intermittent catheterization (CIC), alongside the effective management of urinary tract infections, are critical to the prevention of progressive renal impairments [12]. Chronic kidney disease (CKD), a potential long-term consequence, is associated with elevated levels of systemic oxidative stress, which may exacerbate tissue injury and contribute to sustained inflammatory responses [13,14].

This study aims to evaluate the total antioxidant status (TAS) in patients with NB after MMC, investigate its influence on urinary antioxidant capacity, and examine the correlations with urodynamic parameters, in comparison to those in a reference group.

## 2. Materials and Methods

### 2.1. Patients

The prospective investigation was conducted with 86 children, divided into two groups. The first group constituted 29 children (group 1; median age: 7.83 years; range: 0.5–17) who were operated on for MMC in the neonatal period and diagnosed with NB based on urodynamic investigations. The exclusion criteria for the study group were as follows: NB due to etiologies other than MMC, the presence of an active UTI, or a high detrusor pressure (≥40 cm H_2_O) identified on urodynamic testing, which could independently affect bladder function and outcomes. The urodynamic evaluation in each patient from group 1 was performed using the urodynamic device from Andromeda Medical Systems (Buckinghamshire, UK) placed in our department. The methods, definitions, and units for urodynamic investigation used in this study conform to those of the International Children’s Continence Society [15]. The following parameters were analyzed: detrusor pressure at urgency (Pdet urg), detrusor pressure at the maximum cystometric capacity (Pdet CC), compliance of the bladder wall, and electromyography (EMG) of the sphincter at the beginning (EMG 1) and at the end (EMG 2) of the filling phase. The infusion volume was calculated as the average volume of urine obtained from CIC to estimate bladder function so as to approximate everyday filling and emptying of the bladder in natural conditions best. Active UTIs were excluded on the basis of urinary testing. The lesion level in patients with MMC was determined based on intraoperative findings and radiological assessments and categorized as thoracolumbar, lumbosacral, or sacral. Ambulatory function was evaluated according to Hoffer’s classification [16], which stratifies patients into four groups: non-ambulators, non-functional ambulators, household walkers, and community walkers.

The medical management of NB predominantly involved CIC, which was implemented in 22 out of 29 cases (76%). The pharmacological treatment for NB primarily consisted of antimuscarinic agents, with oxybutynin administered to 16 patients (55%) and tolterodine given to 1 patient (3%). Additionally, doxazosin was prescribed in one case (3%). Notably, 11 of the 29 children (38%) did not receive any form of pharmacotherapy.

The second group comprised 57 healthy children (group 2; median age: 10.1 years; range: 1.5–17.8) who attended routine pediatric visits as part of standard health assessments with no urinary abnormalities or current UTIs, as confirmed by normal urine tests.

We assessed all of the participants’ medical charts to determine their age, sex, anthropometric measurements (weight and height), and renal function parameters: urinary and serum creatinine and serum urea and uric acid. The glomerular filtration rate (GFR) was assessed according to the bedside Schwartz formula: GFR (mL/min × 1.73 m^2^) = 0.413 × height (cm)/serum creatinine (mg/dL).

### 2.2. Biochemistry

Venous blood samples were collected early in the morning (between 8:00 and 9:00) by nursing staff into polypropylene EDTA-containing tubes. Fresh blood was immediately stored on ice at 4 °C. The blood was centrifuged (652× *g* 10 min, 4 °C); the separating serum was carefully collected and stored at −80 °C until further analysis. First morning void spot urine samples were collected for measurement of urinary TAS (uTAS).

TAS was determined in the plasma and urine using an Antioxidant Assay Kit (Cayman Chemical Europe) based on the ability of the antioxidants presents in the sample to inhibit the oxidation of 2,2′-azino-bis(3-ethylbenzothiazoline-6-sulphonic acid (ABTS), which is monitored by reading the absorbance at 660 nm. Urinary creatinine concentrations were used to normalize the TAS measurements in urine to account for the influence of urinary dilution on concentration. Urinary TAS was expressed as the uTAS/creatinine ratio.

### 2.3. Statistics

The data were collected in a Microsoft Excel database. The statistical analysis was performed using Statistica 13.3. (StatSoft Inc., Tulsa, OK, USA). Continuous variables are expressed as medians and ranges, unless stated otherwise. All parameters studied were analyzed using nonparametric tests: the Mann–Whitney and Kruskal–Wallis tests and a Chi2 analysis. Correlations were assessed using Spearman’s test. Correlations were evaluated using Spearman’s rank correlation coefficient. Values of *p* < 0.05 were considered significant.

### 2.4. Ethical Issues

This study was approved by the Ethics Committee of the Medical University of Bialystok, which complies with the World Medical Association’s Declaration of Helsinki regarding ethical conduct in research involving human subjects. Patients and their caregivers were enrolled into this study after informed consent was obtained.

## 3. Results

The characteristics of the children studied are presented in Table 1. The median age of the enrolled patients was 9.25 (0.5–17.8) years. There were no differences in age, gender, or weight, but there were in height. This resulted from the smaller vertebral dimensions and malformations in the bone structure due to MMC in NB group. We found statistically significant differences in the serum and urine creatinine concentrations and the GFR assessed using the bedside Schwartz equation between the studied groups. As shown, the children with NB have a significantly decreased serum TAS and an elevated urinary TAS in comparison with these values in healthy participants.

Among the 29 children with NB, 21 (72%) had a documented history of hydrocephalus, of whom 13 underwent treatment with shunt implantation. The majority of the NB patients, 20/29 (69%), had lumbosacral spinal lesions; 4/29 (14%) had thoracolumbar-level lesions; and 5/29 (17%) had sacral-level lesions. Regarding ambulatory status, as assessed using the Hoffer classification [16], 17/29 (59%) of the children were categorized as non-ambulators or non-functional ambulators, 4/29 (14%) as household walkers, and 8/29 (27%) as community walkers. No statistically significant differences were identified in serum TAS, urinary TAS, or the uTAS/creatinine ratio when they were analyzed in relation to the level of spinal lesions or between the different Hoffer scale groups. Similarly, no significant differences were observed between the catheterized and non-catheterized patients in serum TAS, urinary TAS, or the uTAS/creatinine ratio (*p* = 0.83, *p* = 0.78, and *p* = 0.76, respectively).

Table 2 presents the urodynamic parameters observed in the patients with NB. An analysis of these parameters demonstrated a statistically significant positive correlation between uTAS/creatinine and Pdet CC (r = 0.5, *p* < 0.05). Additionally, a significant negative correlation was identified between uTAS/creatinine and bladder compliance (r = −0.68, *p* < 0.05).

Among the study cohort, only 10 out of 29 patients with NB (34%) exhibited normal bladder wall compliance, defined as greater than 20 mL/cmH_2_O. In this subgroup, the median uTAS/creatinine ratio was 0.06 (range: 0.05–0.11). In contrast, 19 out of 29 patients (65%) demonstrated reduced bladder compliance, defined as less than 10 mL/cmH_2_O, with a median uTAS/creatinine ratio of 0.12 (range: 0.08–0.21). This difference was statistically significant (*p* < 0.001, *p* < 0.05).

## 4. Discussion

To the best of our knowledge, this study is the first clinical evaluation of the serum and urinary TAS in pediatric NB patients after MMC. Its findings are as follows: 1. The serum TAS in the children after MMC was significantly lower than that in the reference group. 2. Urinary TAS and the uTAS/creatinine ratio in NB were significantly higher than those in healthy controls. 3. The uTAS/creatinine ratio correlated positively with detrusor pressure at cystometric capacity and negatively with bladder compliance. The following section will provide a comprehensive, step-by-step analysis of these findings.

The cost common reference range for serum TAS in healthy subjects is 1.0 to 1.8 mmol/L [17]. The median value in NB in our study was 0.31 mmol/L. A low serum TAS signifies that an organism’s ability to neutralize reactive oxygen species (ROS) is compromised, thereby increasing the risk of oxidative damage to the cellular structures and suggesting the presence of oxidative stress. Children with MMC are particularly vulnerable to oxidative stress due to a combination of factors inherent to their condition and its complications, including, e.g., immobilization. Reduced physical activity leads to decreased contraction of the skeletal muscle, resulting in diminished mitochondrial activity and impaired oxygen utilization [18,19]. In our cohort, the majority of the patients with MMC were wheelchair-dependent; however, no statistically significant differences in serum or urinary TAS were observed when they were analyzed according to ambulatory function. This finding suggests that factors beyond reduced mobility may contribute to the observed decrease in serum TAS. Another potential factor may be nutritional deficiency, as feeding difficulties or malabsorption is relatively common in this population and may compromise systemic antioxidant defenses by limiting the availability of essential substances required for the optimal antioxidant enzyme activity [20,21]. Surgical and orthopedic interventions contribute to physiological stress and inflammatory responses that can amplify oxidative damage. Additionally, MMC is frequently associated with hydrocephalus, which often necessitates the placement of a ventriculoperitoneal shunt to manage elevated intracranial pressure. Although the insertion of the shunt effectively alleviates intracranial pressure, it carries inherent risks of neurological injury secondary to shunt malfunction, infection, or over-drainage of cerebrospinal fluid. Neurological damage arising from these complications may contribute further to the development of oxidative stress by promoting inflammatory responses and the excessive generation of ROS [22].

Another complication of MMC is NB, which often leads to recurrent UTIs and chronic inflammation, both of which contribute to the excessive production of ROS [23]. In the present study, we aimed to assess the TAS in the serum and urine from patients with NB to understand their oxidant status better. As there is no standard normal value for the TAS in the urine, these results must be interpreted relative to those for the reference group. Urinary TAS, as well as the uTAS/creatinine ratio, was significantly elevated in NB compared to that in the healthy controls. This observation may indicate adaptive upregulation of the antioxidant defenses in response to increased oxidative stress.

As the majority of our NB patients had neurogenic detrusor overactivity, it is important to emphasize that the pathophysiology of overactive bladder is multifactorial, with excessive oxidative stress recognized as one of the contributing mechanisms [24,25]. The neurogenic detrusor overactivity in our cohort was effectively managed using anticholinergic therapy. Elevated bladder storage pressures are known to increase the risk of deterioration of the upper urinary tract; therefore, reducing this pressure is a primary therapeutic objective [26]. A key indicator of effective treatment in our cohort was that Pdet CC remained within the normal range and did not exceed 40 cm H_2_O. Furthermore, commonly used clinical markers of renal function, including creatinine, urea, uric acid, and the Schwartz GFR, remained within the norm. Despite this, we observed an increase in uTAS, suggesting that oxidative imbalance persists even when conventional measures of renal function appear normal. Additionally, we evaluated the urodynamic findings and examined their correlation with TAS. We found that the uTAS/creatinine ratio correlated positively with Pdet CC and negatively with bladder compliance. These findings imply that oxidative stress may contribute to adverse urodynamic profiles, characterized by elevated bladder pressures and impaired bladder compliance, potentially placing the upper urinary tract at risk of long-term deterioration. It may be proposed that in clinical practice, adopting Pdet CC at a lower threshold than the conventional 40 cm H_2_O norm could be beneficial for this specific patient population.

Understanding the pathological role of oxidative stress within the bladder is crucial to the development of potential therapeutic strategies. Animal models of spinal cord injury have shown improvements in bladder function following antioxidant treatment [27]. Several studies have demonstrated that antioxidant therapy represents a promising approach to reducing ROS levels in chronic kidney disease [28]. Limited studies exist, but some small-scale trials and translational research suggest its therapeutic potential [29,30,31].

Our study offers several notable advantages. The major one is that it represents the first investigation of the TAS in pediatric NB. Secondly, we measured the TAS in both serum and urine samples. It is important to highlight that blood collection can induce significant stress in pediatric patients, potentially affecting the outcomes measured. In contrast, urine collection is inexpensive, rapid, and noninvasive, thereby minimizing the likelihood of artificially elevated oxidative stress markers during sample acquisition. Furthermore, the assessment of TAS in the urine remains relatively underexplored, with only a limited number of clinical studies investigating urinary markers of oxidative stress in the context of kidney diseases. Additionally, our study had the opportunity to assess urodynamic parameters and investigate their correlation with TAS. The relatively small sample size represents a limitation of our study, although it reflects the low prevalence of MMC. Future research involving larger cohorts is essential to confirm and strengthen our results.

## 5. Conclusions

This study is the first to clinically evaluate the serum and urinary TAS in pediatric patients with NB due to MMC. The findings reveal a marked reduction in serum TAS, indicating systemic oxidative stress, alongside elevated urinary TAS and uTAS/creatinine ratios, which may reflect a compensatory response to localized oxidative challenges within the bladder. Notably, the positive correlation between the uTAS/creatinine ratio and detrusor pressure, as well as the negative correlation with bladder compliance, suggest that oxidative stress may contribute to impaired urodynamic function. These observations underscore the potential clinical value of incorporating oxidative stress markers into the assessment and management of NB and support further exploration of antioxidant-based therapeutic strategies.

## Figures and Tables

**Table 1:** The demographic characteristics of the patients with NB and the reference group. GFR, glomerular filtration rate; TAS, total antioxidant status; uTAS, urinary total antioxidant status; * *p* < 0.05.

Variables	Group 1 *n* = 29	Group 2 *n* = 57	*p* Value
Gender: Female/male *n* (%)	12(41)/17(59)	31(54)/26(46)	0.71
Median (minimum–maximum)			
Age (years)	7.83 (0.5–17)	10.1 (1.5–17.8)	0.14
Height (cm)	135 (70–167)	152.5 (89–180)	0.04 *
Weight (kg)	25 (6.9–92)	34.5 (10–84)	0.16
Serum creatinine (mg/dL)	0.32 (0.19–0.77)	0.5 (0.2–1.07)	<0.001 *
Urinary creatinine (mg/dL)	51.6 (14–111)	100 (23.5–315)	<0.001 *
Urea (mg/dL)	26 (11–42)	28 (15–43)	0.28
Uric acid (mg/dL)	3.89 (2.76–5.7)	4.08 (2.46–7.09)	0.18
eGFR (bedside Schwartz) (mL/min/1.73 m^2^)	154.2 (89.6–247.8)	121.8 (92.5–247.8)	<0.001 *
Serum TAS (mmol/L)	0.31 (0.04–5.6)	1.46 (0–11.98)	<0.001 *
uTAS (mmol/L)	8.81 (1.94–29)	4.51 (1.01–24.9)	<0.001 *
uTAS/creatinine ratio	0.1 (0.01–1.2)	0.04 (0.008–0.09)	<0.001 *

**Table 2:** Urodynamic parameters of patients with NB. Pdet urg, the detrusor pressure at urgency; Pdet CC, the detrusor pressure at the maximum cystometric capacity; EMG 1, electromyography of the sphincter at the beginning of the filling phase; EMG 2, the former at the end of the filling phase.

Urodynamic Parameters	
Pdet urg (cmH_2_O)	30 (10–80)
Pdet CC (cmH_2_O)	10 (2–34)
MaxCC (mL)	150 (38–287)
Compliance (mL/cmH_2_O)	12 (0.3–70)
EMG 1 (mV)	3 (0–25)
EMG 2 (mV)	5 (0–30)

## Data Availability

The data presented in this study are available on request from the corresponding author. The data are not publicly available for ethical and privacy reasons.

## Abbreviations

The following abbreviations are used in this manuscript:

NB	neurogenic bladder
MMC	myelomeningocele
GFR	glomerular filtration rate
ABTS	2,2′-azino-bis(3-ethylbenzothiazoline-6-sulphonic acid
TAS	total antioxidant status
uTAS	urinary total antioxidant status
UTI	urinary tract infections
CIC	clean intermittent catheterization
CKD	chronic kidney disease
Pdet urg	the detrusor pressure at urgency
Pdet CC	the detrusor pressure at the maximum cystometric capacity
EMG	electromyography
EMG1	electromyography of the sphincter at the beginning of the filling phase
EMG2	electromyography of the sphincter at the end of the filling phase
ROS	reactive oxygen species
